# Surface properties of a new lithium disilicate glass-ceramic after grinding

**DOI:** 10.1007/s10856-021-06592-w

**Published:** 2021-08-28

**Authors:** Larissa Natiele Miotto, Mariana de Oliveira Carlos Villas-Bôas, Edgar Dutra Zanotto, Eduardo Bellini Ferreira, Laiza Maria Grassi Fais, Lígia Antunes Pereira Pinelli

**Affiliations:** 1 Department of Dental Materials and Prosthodontics, School of Dentistry of Araraquara, São Paulo State University (UNESP), Araraquara, SP Brazil; 2 Department of Materials Engineering, Vitreous Materials Laboratory, Federal University of São Carlos (UFSCar), São Carlos, SP Brazil; 3 Department of Materials Engineering, São Carlos School of Engineering, University of São Paulo (USP), São Carlos, SP Brazil

## Abstract

This study aimed to evaluate the effect of grinding on some surface properties of two lithium disilicate-based glass-ceramics, one experimental new product denominated LaMaV Press (UFSCar-Brazil) and another commercial known as IPS e-max Press (Ivoclar), in the context of simulated clinical adjustment. Discs (*N* = 24, 12 mm in diameter) were separated into four groups: LaMaV Press with no grinding (E), LaMaV Press after grinding (EG), IPS e-max Press with no grinding (C), and IPS e-max Press after grinding (CG). A 0.1-mm deep grinding was carried out on EG and CG samples (final thickness of 1.4 mm) using a diamond stone in a low-speed device. The E and C samples had the same thickness. The effect of grinding on the sample surfaces was evaluated by X-ray diffraction, mechanical and optical profilometry, scanning electron microscopy, goniometry, and Vickers hardness. The mean roughness (Ra) was evaluated by Kruskal–Wallis and Student–Newman–Keuls statistics. The surface energy (SE) by the sessile drop method and Vickers hardness (VH) were analyzed using two-way ANOVA. The Ra medians were E = 1.69 µm, EG = 1.57 µm, C = 1.45 µm, and CG = 1.13 µm with *p* = 0.0284. The SE and VH were similar for all materials and treatments. Grinding smoothed the surfaces and did not significantly alter the hardness and surface energy of both LaMaV Press and IPS e-max Press. These glass-ceramics presented similar surface properties, and clinical adjustments can be implemented without loss of performance of both materials.

**Figure:**
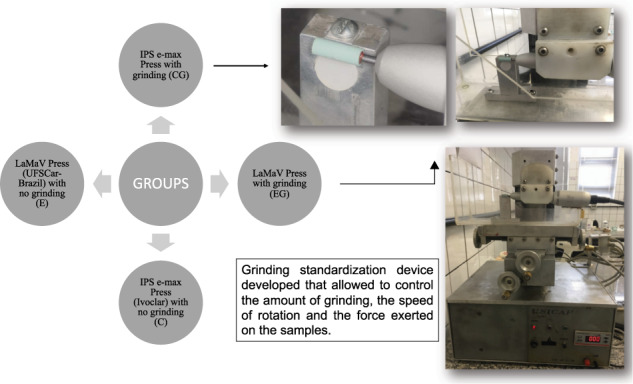
A grinding standardization device developed that allowed to control the amount of grinding, the speed of rotation speed and the force exerted on the samples.

A grinding standardization device developed that allowed to control the amount of grinding, the speed of rotation speed and the force exerted on the samples.

## Introduction

Lithium disilicate (LS2) glass-ceramics were introduced in dentistry 20 years ago [1]. When the parent glass of these materials is conveniently heated, a lithium phosphate nanophase (Li_3_PO_4_) precipitates and acts as a catalyst for the heterogeneous nucleation of lithium metasilicate (LS), concurrently with the formation of LS2 crystals [2]. As a result, the final microstructure comprises ~70 vol% crystalline phases, being LS2 the primary phase [3–5].

These LS2 glass-ceramics are produced by melting a mixture of chemicals and quenching it to form a glass, which is subsequently heat-treated for controlled crystallization [3]. First, a precursor material consisting of partially crystallized glass is heated to achieve a suitable viscosity and injected into a die in a class of such glass-ceramics formed by injection molding. Then, it is cooled down to some extent, but kept above its glass transition for further crystallization.

Some commercial LS2 glass-ceramics achieve a fracture strength close to 400 MPa [3], which despite being very high, it is still not enough for prostheses with more than three units [6, 7]. However, changes in composition and heat treatment could result in microstructures with improved mechanical properties, as demonstrated by Höland et al. [8].

A research effort for developing new LS2-based glass-ceramics of this class was performed at the Vitreous Materials Laboratory (LaMaV) at the Federal University of São Carlos (DEMa/UFSCar), aiming to improve their microstructure and properties. The pursued novelty refers to microstructure optimization based on the previously determined crystallization kinetics of glasses with different chemical compositions, similar crystal phases, and tailored internal residual stresses to maximize mechanical properties. The compositions and expected microstructures were designed using homemade software (Reformix 3.0), combined with interactive experiments employing differential scanning calorimetry and scanning electron microscopy (SEM). Approximately 50 formulations and hundreds of thermal treatments were tested. Some of these new LS2-based materials can be hot-pressed or processed by a CAD/CAM machining for application as dental prosthesis [9, 10].

Adjustment by grinding with abrasive tools is a routine procedure for dentists when adapting prosthesis cores [4, 11–13], or aiming to gain space for a porcelain veneer, occlusal adjustment, endodontic access, or crown removal [14]. However, grinding procedures often cause significant surface modifications [4] that alter the strength and reliability of the material, depending on whether they increase or decrease the surface roughness, thus introducing or removing cracks or other defects, or generating residual stresses [4, 5, 11–13, 15–19]. Therefore, the effect of clinical adjustments on the performance of hot-pressed LS2 glass-ceramics must be evaluated.

If the grinding procedure decreases the material roughness, its strength may increase [12, 17–19]. Although a regular finishing may also increase the product reliability that does not mean that the strength will also increase [4, 5, 11, 12, 17–19]. A more homogeneous topography between the core and the porcelain veneer ensures a smoother interface with fewer defects [4, 17]. Nonetheless, this interface cannot be excessively polished, as it would impair the bonding between materials [20], causing cracks that increase delamination incidence [4, 13]. In addition to roughness, the surface energy (SE) may also affect the contact angle between the glass-ceramic core and the veneer [20]. As the vitreous phase is generally softer and weaker than the crystal phase [20–24], grinding may selectively remove the vitreous phase, altering the SE of the material [22]. Hence, characterize the SE effect may be relevant on the evaluation of the core/veneer interface strength [20].

Additionally, the core hardness interferes with the material cutting procedure, being an essential factor that deserves attention. In other words, the harder the material, the greater the force needed to cut it [25] and the shorter the lifetime of the burs [26]. Independent of the diamond bur selected, the process to cut an LS2 glass-ceramic block requires a significant amount of time [14]. Material hardness is also important when considering different materials for ceramic prostheses, such as nano- and hybrid composites, which may cause enamel wear of the antagonist tooth [27].

Therefore, the evaluation of a dental glass-ceramic response to external surface grinding is crucial for clinical adjustment procedures [16]. In this work, we analyze the effect of surface grinding on the hardness, roughness, surface topography, crystalline phases, and SE of an experimental LS2 glass-ceramic (LaMaV Press, UFSCar), and compared it with the commercial material IPS e-max Press (Ivoclar) as a benchmark. The null hypothesis is that grinding does not alter the surface properties and Vickers hardness (VH) of these glass-ceramics.

## Materials and methods

The LaMaV Press LS2 glass-ceramic (*n* = 12) (composed of Sb_2_O_3_, K_2_O, MgO, CaO, BaO, ZnO, Al_2_O3, ZrO_2_, Na_2_O, P_2_O_5_, Li_2_O, and SiO_2_) was supplied as injected discs (1.5 mm in thickness × 12 mm in diameter). The IPS e-max Press (Ivoclar Vivadent, Schaan, Liechtenstein) was adopted as the control group (*n* = 12).

To obtain the IPS e-max Press specimens, commercial ingots were injected according to the manufacturer’s recommendations. Wax discs with specimen dimensions were embedded in refractory material molds (Gilvest Hs, Bradent, Cotia, SP, Brazil), and subsequently heated in a furnace (EDG 3000, EDG, São Carlos, SP, Brazil) for ring heating, coating expansion, and wax elimination. Next, the glass-ceramic injection was carried out in an oven with preset parameters (Programat EP3000, Ivoclar Vivadent, Schaan, Liechtenstein), followed by cooling and divestment aided by sandpaper and blasting with glass beads.

Afterwards, all specimens were normalized by polishing with SiC sandpaper from #100 to #1500 (401Q, 3M ESPE, Sumaré, SP, Brazil) in an automatic metallographic machine (Aropol 2V, Arotec Indústria e Comércio, Cotia, SP, Brazil) at 600 rpm, with constant water irrigation. The sample dimensions were measured using a digital caliper (500–144B, Mitutoyo Sul Americana, Suzano, SP, Brazil), accepting a variation of 0.01 mm. Discs of 12 mm in diameter and 1.4 mm in thickness for the “without-grinding” groups and 12 mm by 1.5 mm for the “with-grinding” groups (Table 1) were used as test specimens. The analysis surfaces were abraded by compressed air with Al_2_O_3_ powder of 120-μm medium diameter at 2 bars for 20 s and at a constant distance of 10 mm. Finally, the specimens were cleaned in an ultrasonic bath for 5 min in distilled water, followed by 5 min in isopropyl alcohol, and then dried in air.

**Table 1:** Nomenclature adopted for the samples and conditions evaluated

Sample	Condition	Variable	
		Without grinding	With grinding
IPS e-max Press	Control group	C	CG
LaMaV Press	Experimental group	E	EG

The test grinding was then performed in a standard machine at 20,000 rpm under 100 gf. A depth of 0.1 mm was removed from the upper surface of the EG and CG specimens with the aid of a cylindrical diamond stone (MasterCeram^®^, MCE 133 104, Eurodental, São Paulo, SP, Brazil) attached to a low-speed electric handpiece (LB 100, Beltec, Araraquara, SP, Brazil). It is worth mentioning that dental technicians and dentists use this diamond stone to adjust the surface of LS2 copings for its high abrasion power.

One disc of each group was analyzed by X-ray diffractometry (RINT2000, RIGAKU, São Paulo, SP, Brazil) using Cu-Kα radiation, 2θ between 10° and 80°, an angular step of 0.02°, and continuous scan at 3°/min. Additionally, the crystalline phases were identified with the ICSD-PDF + 2 database and quantitatively evaluated by the Rietveld method [28] aided by the Topas Academic software [29].

The VH (GPa) was measured in a Micromet 2100 hardness tester (Buehler, Lake Bluff, Illinois, USA) with a load of 500 gf for 15 s. Six specimens per group were analyzed and six measurements were performed in each sample. The mean roughness (Ra, µm) was measured in a profilometer (Mitutoyo SJ 400, Mitutoyo Corporation, Yokohama, Kanagawa, Japan) with an accuracy of 0.01 μm, full scale of 2.5 mm, active tip velocity of 0.5 mm/s, and radius of the active tip of 5 μm. Six specimens per group were analyzed, with three measurements for each surface. The mean values for VH and Ra were then determined.

The surface topography of the specimens was evaluated by an optical confocal profilometer (PB1000, Nanovea, USA) in an area of 0.5 mm × 0.5 mm and a spot size of 3 µm, according to ISO 25178. For the surface morphology characterization, one specimen per group had its surface coated with platinum, followed by analysis in a SEM (Inspect F50, FEI, Achtseweg Noord 5, Eindhoven, the Netherlands) with a weak beam voltage of 3 kV.

The SE (mN/m) was determined by measuring the static contact angle in six specimens per group. The contact angle was determined by the sessile drop method in an automatic goniometer (Dataphysics, OCA20, Filderstadt, Baden- Württemberg, Germany). Liquids with different polarities (water, glycerol, and diiodomethane) were used as reference. The contact angle was determined at a controlled temperature (20 °C) after a stabilization time of 30 s. The mean contact angle for each liquid was calculated from three readings performed on the surface of each sample with intervals of 1 week between the measurements. The SE was calculated based on Young’s equation (1):

$$\gamma _S - \gamma _{SL} - {{{{{\mathrm{{\Pi}}}}}}}_{eL} = \gamma _L\cos \theta _L(1),$$

where *γ*_*S*_ is the solid-air SE, *γ*_*SL*_ is the solid-liquid interfacial energy, *γ*_*L*_ is the liquid-air SE, II_*eL*_ is the pressure of the liquid film, and *θ*_*L*_ is the contact angle. *γ*_*SL*_ and II_*eL*_ were supplied by the software.

The resulting data were statistically analyzed using the Biostat 5.1 software at a significance level of 5%. All data were submitted to a normality test by Shapiro–Wilk and homoscedasticity by Student’s *t* test. The SE and VH were analyzed using two-way ANOVA. The Ra was evaluated using the Kruskal–Wallis test followed by the Student–Newman–Keuls test.

## Results

**Fig. 1:**
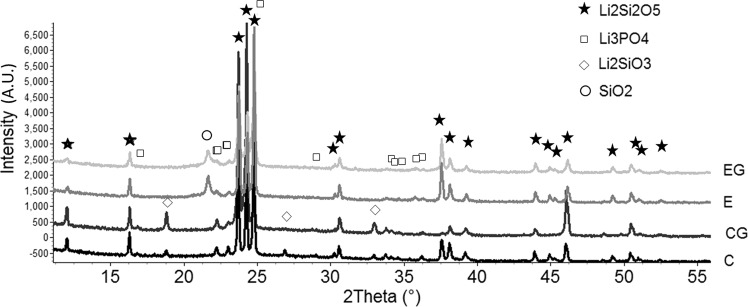
X-ray diffraction patterns for groups C, CG, E and EG

**Fig. 2:**
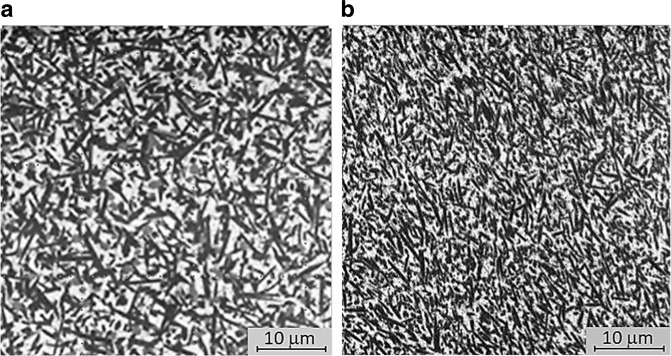
SEM microstructures of polished and sandblasted surfaces of **a** LaMaV Press and **b** IPS e-max Press

The XRD results are shown in Fig. 1 and Table 2. It can be observed that within the error limits the residues in Table 2 approach zero. There is no minor crystalline phase for a good approximation besides those values shown, which could impair the results presented below. The resulting microstructures are displayed in Fig. 2, where it is possible to see crystals embedded in a residual glass, whose volume fractions were not quantified. Even though the main crystalline phase has a lathed shape in both materials, the crystals in the LaMaV Press are larger, randomly oriented, and with a broader size distribution than those in the IPS e-max Press, which are partially aligned.

**Table 2:** Quantitative phase analysis for groups C, CG, E and EG

Group	Li_2_Si_2_O_5_ (%)	Li_3_PO_4_ (%)	Li_2_SiO_3_ (%)	SiO_2_ (%)
C	85.4 (7)	9.8 (6)	4.8 (3)	–
CG	78.4 (8)	11.8 (8)	9.8 (4)	–
E	85.2 (8)	5.4 (6)	–	8.6 (4)
EG	82.3 (3)	8.5 (2)	–	7.7 (5)

The standard deviation of the measurements is shown in parentheses (mass%)

The IPS e-max Press groups C and CG presented XRD peaks corresponding to lithium disilicate (Li_2_Si_2_O_5_), lithium phosphate (Li_3_PO_4_), and lithium metasilicate (Li_2_SiO_3_), confirming the well-known results for this material. In contrast, the LaMaV Press groups E and EG showed XRD peaks associated with Li_2_Si_2_O_5_, Li_3_PO_4_, and cristobalite (SiO_2_). Therefore, it can be observed that both IPS e-max Press and LaMaV Press presented two similar crystalline phases, Li_2_Si_2_O_5_ and Li_3_PO_4_, with major amounts of the first, which despite diminishing after grinding, it still appeared in a greater extension in the IPS e-max Press. Although the diffractograms look similar (Fig. 1), cristobalite was only observed in the E and EG specimens, whereas Li_2_SiO_3_ was only detected in the C and CG specimens.

The different phases in IPS e-max Press and LaMaV Press support the diverse nature of these glass-ceramics. The surface grinding affects the proportion of surface phases differently, possibly causing selective phase removal as a function of the nature and ratio of residual glass, residual stresses, elastic modulus, ductility, and hardness of the different phases. The precise evaluation of such factors is not the task of this paper, yet this selective wearing must be confirmed in future works to explain veneering adhesion.

The VH (GPa) values were as follows: C = 5.6 ± 0.3; CG = 5.7 ± 0.4; E = 5.9 ± 0.2, and EG = 5.7 ± 0.5. Table 3 shows the corresponding two-way ANOVA statistical test. The VHs of the groups (both LaMaV Press and IPS e-max Press) were found to be equal (*p* = 0.22). The grinding did not change the VH of any product (*p* = 0.88). There was no interaction between the groups (*p* = 0.48).

**Table 3:** Results of the ANOVA two-way statistical test of VH

Source of variation	SQ	gl	MQ	*F*	*p*
Brand	2020	1	2021	1.60	0.22
Treatment	28	1	28	0.02	0.88
Interactions	633	1	633	0.50	0.48
Total	22,827	19			

**Fig. 3:**
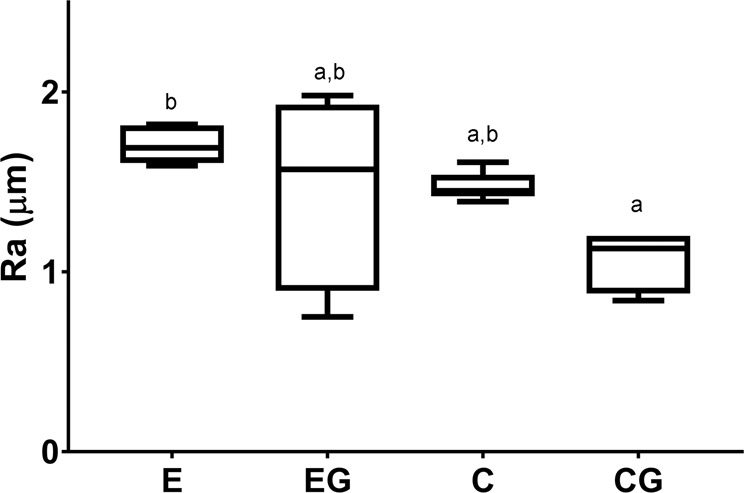
Box plot graphic of median roughness (Ra, in μm). Different lowercase letters denote significant differences among the groups

Figure 3 shows the roughness medians. The Kruskal–Wallis test identified some differences among the groups (*p* = 0.028). The Ra (µm) medians were E = 1.69, EG = 1.57, C = 1.45, and CG = 1.13. The Student–Newman–Keuls test did not indicate any significant difference between groups C and E. The after-grinding roughness medians of both materials were equal, yet with a greater variation between the maximum and minimum roughness in the EG. Grinding did not alter the Ra inside the groups.

**Fig. 4:**
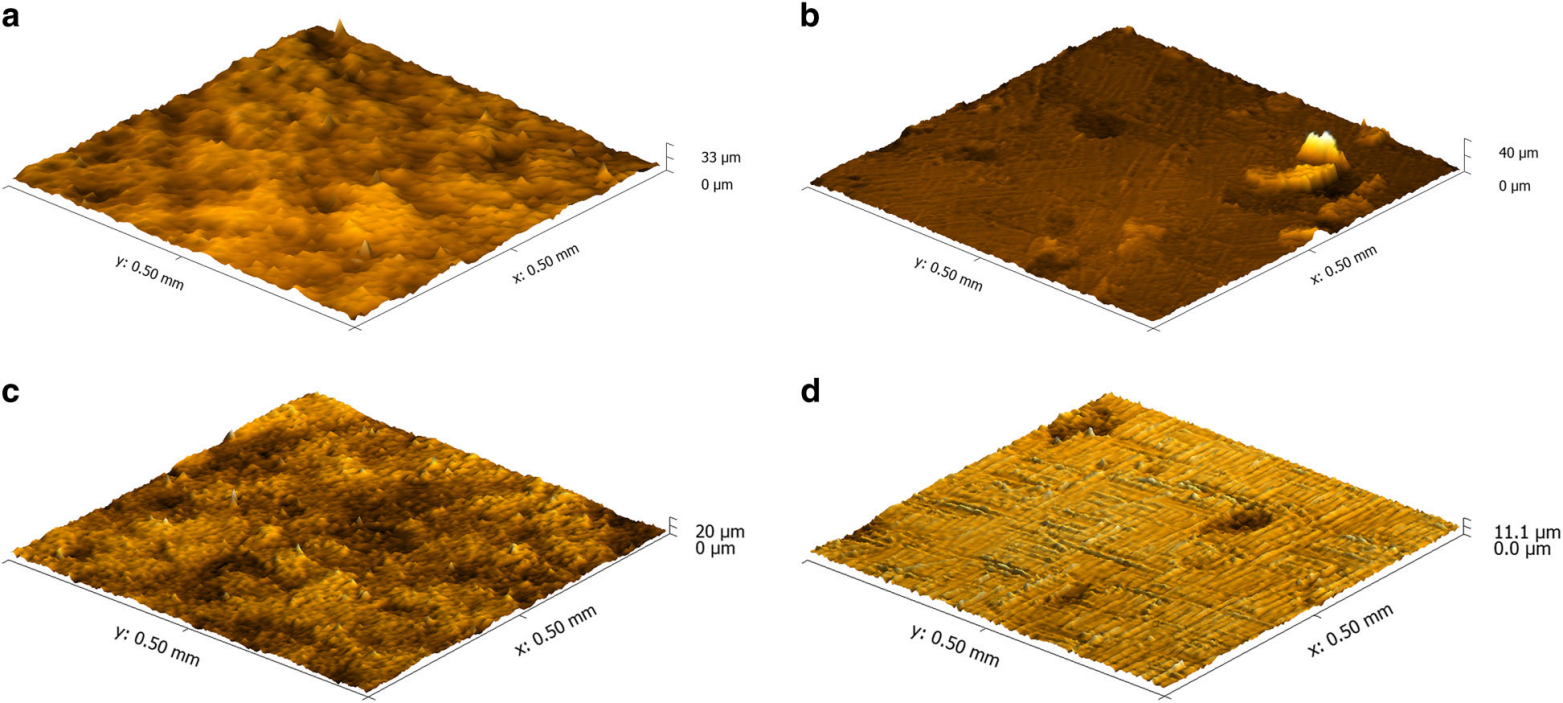
Three-dimensional surface images corresponding to the experimental groups: **a** group C, **b** group CG, **c** group E, **d** group EG

Figure 4 shows the three-dimensional surface images obtained by optical confocal profilometry. Groups C and E (Fig. 4a, c, respectively) presented irregular surfaces with a notable difference between peaks and valleys, but without visual differences between them. On the other hand, the CG and EG ground samples (Fig. 4b, d, respectively) were smooth with some grooves on the surface the grinding procedure.

**Fig. 5:**
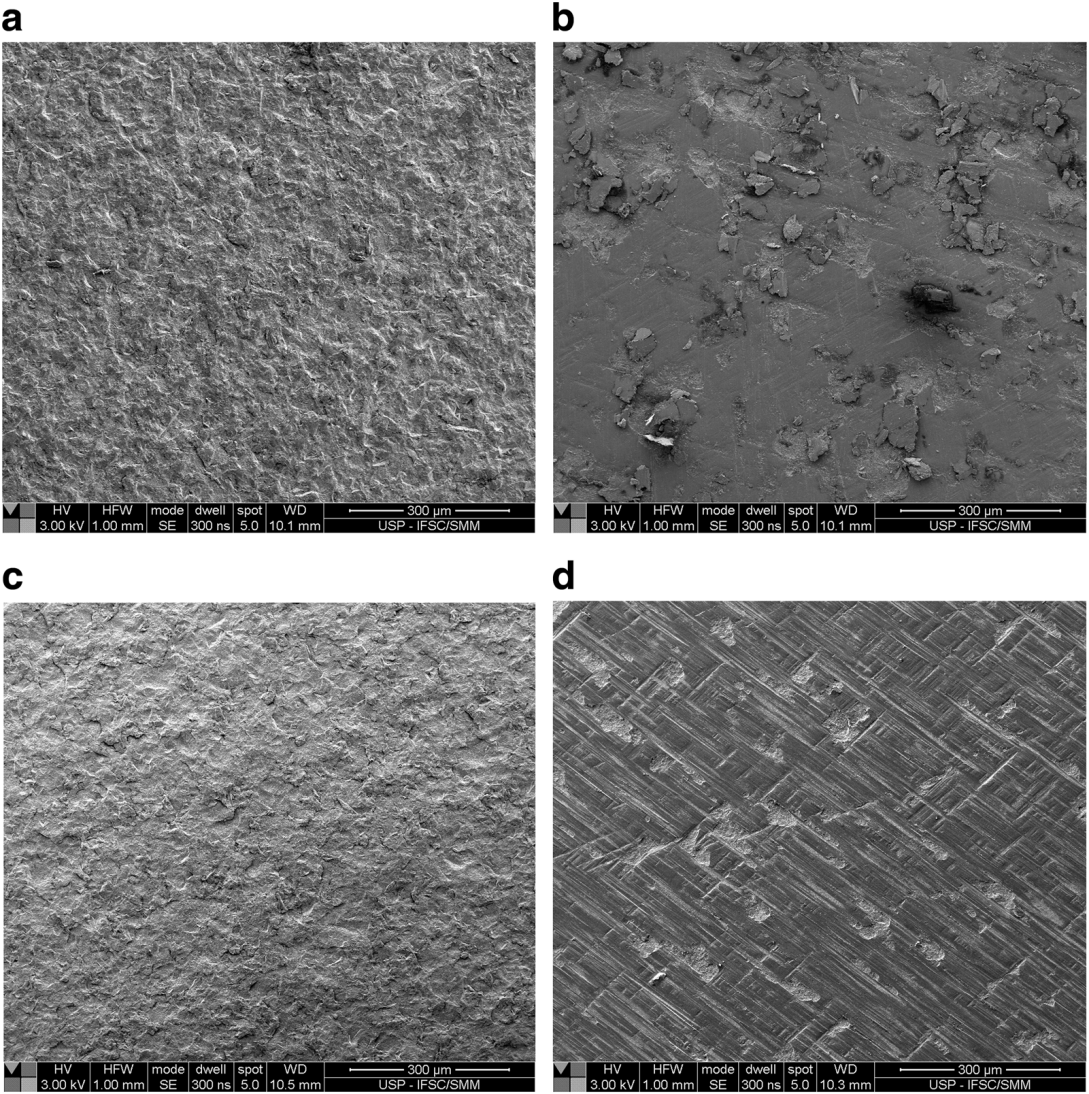
Surface microstructure corresponding to the experimental groups: **a** group C, **b** group CG, **c** group E, **d** group EG (300-μm scale bars)

Figure 5 shows the surface images obtained by SEM. The irregular and rough surface morphologies in Fig. 5a, c are similar and compatible with the profilometry results. After grinding, the surfaces became flattered with some scratches (Fig. 5b, d for CG and EG, respectively). The resulting grooves on the EG samples (Fig. 5d) were more evident and typical of the cutting tool than the CG (Fig. 4b), which can improve its adhesion to a porcelain veneer. The tracks in Fig. 5b (CG) were smoother than those in Fig. 5d (EG).

The mean and standard deviation of the SE (mN/m) were C = 47 ± 9, CG = 56 ± 14, E = 58 ± 10, and EG = 44 ± 8. The two-way ANOVA statistical test (Table 4) revealed no significant differences between the experimental and commercial LS2 glass-ceramics. Thus, the grinding procedure did not cause a significant change in the SE at room temperature.

**Table 4:** Results referring to the ANOVA two-way statistical test for SE

Source of variation	SQ	gl	MQ	*F*	*p*
Brand	1.39	1	1.39	0.013	0.910
Treatment	41.18	1	41.18	0.39	0.539
Interactions	680.24	1	680.24	6.49	0.051
Total	2398.36	19			

## Discussion

Adjustment by grinding with abrasive tools is a routine procedure for dentists and dental technicians when adapting implant cores [4, 11–13]. Although the rectification procedure can cause significant superficial changes [4], impacting the prosthesis performance, the evaluation of its effect in the literature is controversial and scarce. Therefore, it is essential to assess the behavior of LS2 glass-ceramics after grinding during clinical adjustment.

As diamond stones are known to promote less aggressive grinding than burs, a smooth surface was expected. In previous studies [30, 31], a diamond stone similar to the one used in this work yielded a smooth surface when grinding a previously sanded zirconia. However, glass-ceramics based on lithium disilicate have not been fully evaluated yet. In this work the initial sample surfaces were normalized with sandpaper, followed by sandblasting. Afterwards, the grounded groups were subjected to grinding with a diamond stone. Unground control groups were used as reference.

The effect of grinding the outer surface of two LS2-based glass-ceramics (LaMaV Press and IPS e-max Press) was evaluated by probing their crystalline phases, VH, and surface properties. The null hypothesis that grinding with a diamond stone does not alter these materials differently was accepted.

XRD analyses revealed that both materials are mainly composed of crystalline Li_2_Si_2_O_5_, which agrees with other authors in the case of IPS e-max Press [1, 32–34]. The IPS e-max Press also presented Li_3_PO_4_ and Li_2_SiO_3_, as observed in the literature [1, 33, 34]. The amounts of Li_2_Si_2_O_5_ and Li_3_PO_4_ in both groups (Table 2) were very close. However, a silica polymorph (SiO_2_) was observed in the LaMaV Press, but not lithium metasilicate. The different minor crystalline phases are due to differences in the materials composition and processing.

Figure 1 shows that some peaks (2*θ* = 37°, 45°, and 49.2°) corresponding to Li_2_Si_2_O_5_ (LS2) vanished in the ground IPS E-max Press (CG sample). At the same time, the intensity of other Li_2_Si_2_O_5_ peaks increased (e.g., at 46°), indicating a texture caused by grinding. In Table 2, it can be observed that the Li_2_Si_2_O_5_ weight percentage decreased from 85.4 ± 0.7 to 78.4 ± 0.8, whereas an increase was noted in the percentages of Li_3_PO_4_ (9.8 ± 0.6 to 11.8 ± 0.8) and Li_2_SiO_3_ (4.8 ± 0.3 to 9.8 ± 0.4). Such change can be supposedly assigned to the selective grinding of Li_2_Si_2_O_5_ grains in the CG surface, whose crystallographic orientations relative to the diffracting X-ray beam correspond to cleavage planes that fracture easily. This phenomenon did not happen with the same intensity in the ground LaMaV Press (EG sample), probably due to its larger grains and misaligned grains, which could prevent cleavage. The confirmation of this hypothesis, together with its effect on long-term chemical and mechanical properties, is proposed as future work.

Cutting may be difficult, depending on the hardness of the material. High hardness also increases the wear of both cutting tools [24, 25] and the antagonist tooth. Song et al. [16] reported a VH of 5.5 GPa for the IPS e-max Press, which was close to the values obtained in this work (5.6–5.9 GPa). Although the VH of the LaMaV Press was ~5.9 GPa, an statistical analysis (Table 3) did not show any significant difference between the LaMaV Press and IPS e-max Press glass-ceramics.

Furthermore, since grinding is not expected to alter the VH in the present case, it is possible to infer that both materials present similar behavior concerning clinical adjustment procedures. It is important to mention that the hardness of LS2 glass-ceramics was found to be much lower than that of zirconia [35] and closer to that of tooth enamel, which could result in less wear of the cutting tools and the antagonist tooth, making LS2 glass-ceramics an interesting material for restorative dentistry.

Regarding the roughness results, we verified that both glass-ceramics were not different after grinding. The initial Ra (E = 1.6 μm and C = 1.45 μm) values after sandblasting were close to those found by Albakry et al. [11]. However, literature results [4, 5, 11] indicate that the after-grinding roughness of LS2 glass-ceramics is lower than the value observed in this study, probably because such studies used sandpaper instead of a diamond stone.

Although no protocol establishes the roughness for dental prostheses, IPS e-max Press was used as a benchmark due to its well-accepted performance in this application. The LaMaV Press roughness was similar to that of the IPS e-max Press, indicating that it would behave likewise in dental prostheses. Even though the mean roughness of both materials was equal within the error limits (CG = EG), the interval between the maximum and minimum values was larger for the LaMaV Press EG, which may promote better adhesion to a porcelain veneer.

The three-dimensional surface images obtained by optical confocal profilometry and SEM showed similar initial surface morphologies for both materials. In these images, rough surfaces with a notable difference between peaks and valleys were initially observed (Figs. 4a, c and 5a, c), corroborating studies of other authors [4, 5, 11]. Grinding caused surface rectification and elimination of rugosity peaks, as observed by Wang et al. [4], with preservation of some grooves in different directions. It is known that grinding produces a complex topography and roughness on the surface, causing a defect distribution that may affect adhesion to a bilayer composite [4]. In general, reducing the interface roughness allows better stress distribution, thus improving ceramic fracture strength and reliability. However, too smooth surfaces tend to decrease adhesion and increase the incidence of veneer delamination [4, 13, 17, 20].

Previous studies indicated that surface roughness influences the contact angle and the topography, affecting the SE [20–24]. The higher the veneer ability to wet and flow in the irregularities of the core surface, the higher the compatibility and adhesion between them [22, 24]. However, herein the SE did not vary for both LS2 glass-ceramics with grinding, as no significant variation in roughness occurred. Therefore, the grinding operation used in this work probably does not physically modify the compatibility between the veneer and LS2 glass-ceramic cores.

The obtained results suggest that the LaMaV Press glass-ceramic has surface properties and hardness similar to those of the IPS e-max Press. Furthermore, it was shown that these properties are not significantly affected by grinding with a diamond stone. However, to better explore the grinding of LS2 glass-ceramics, future studies are needed, for example, to evaluate their effect on the fracture strength and long-term mechanical and chemical resistance as a function of different process variables and diamond burs.

The microstructure of the LaMaV Press glass-ceramic was developed for dental applications [9] by adjusting its chemical composition and thermal treatment, as well as controlling its crystal size distribution, fraction, morphology and internal residual stresses. As a result, the LaMaV Press material has larger crystals with broader size distribution and more randomly oriented than the IPS e-max Press, in which the crystals line up to some extent. Moreover, this work shows that both materials present similar performances after grinding for dental prosthesis adjustment.

## Conclusion

Grinding with a diamond stone did not significantly alter the surface properties of both LaMaV Press and IPS e-max Press LS2 glass-ceramics. Additionally, the LaMaV Press showed surface properties similar to those of the commercial IPS e-max Press. It was then concluded that clinical adjustment by grinding can be performed with a diamond stone without loss of performance of both materials.

## References

[1] Lien W, Roberts HW, Platt JA, Vandewalle KS, Hill TJ, Chu TM (2015). Microstructural evolution and physical behavior of a lithium disilicate glass-ceramic. Dent Mater.

[2] Höland W, Apel E, Van’t Hoen C, Rheinberger V (2006). Studies of crystal phase formation in high-strength lithium disilicate glass-ceramics. J Non-Cryst Solids.

[3] Ivoclar Vivadent. IPS e-max lithium disilicate: The Future of All-Ceramic Dentistry. 2009. p. 2–5. https://www.thaidentalcenter.com/img/treatments/veneers/EmaxMaterialScience.pdf. Acessed Nov 2013.

[4] Wang XD, Jian YT, Guess PC, Swain MV, Zhang XP, Zhao K (2014). Effect of core ceramic grinding on fracture behaviour of bilayered lithium disilicate glass-ceramic under two loading schemes. J Dent.

[5] Vrochari AD, Petropoulou A, Chronopoulos V, Polydorou O, Massey W, Hellwig E (2015). Evaluation of surface roughness of ceramic and resin composite material used for conservative indirect restorations after repolishing by intraoral means. J Prosthodontics.

[6] Höland W, Rheinberger V, Apel E, Ritzberger C, Eckert H, Mönster C (2007). Mechanisms of nucleation and crystallisation in high strength glass-ceramics Physics and Chemistry of Glasses. Eur J Glass Sci Technol Part B.

[7] Serbena FC, Zanotto ED (2012). Internal residual stresses in glass-ceramics: a review. J Non-Cryst Solids.

[8] Höland W, Rheinberger V, Apel E, van’t Hoen C (2007). Principles and phenomena of bioengineering with glass-ceramics for dental restoration. J Eur Ceram Soc.

[9] Villas-Bôas MOC. Development and characterization of glass-ceramics of the Li_2_O-SiO_2_ system for dental application. Ph.D. Thesis. São Carlos: UFSCar; 2013 (in Portuguese).

[10] Villas-Boas MOC, Serbena FC, Soares VO, Mathias I, Zanotto ED (2019). Residual stress effect on the fracture toughness of lithium disilicate glass-ceramics. J Am Ceram Soc.

[11] Albakry M, Guazzato M, Swain MV (2004). Effect of sandblasting, grinding, polishing and glazing on the flexural strength of two pressable all-ceramic dental materials. J Dent.

[12] Fleming GJ, El-Lakwah SF, Harris JJ, Marquis PM (2004). The influence of interfacial surface roughness on bilayered ceramic specimen performance. Dent Mater.

[13] Fleming GJ, Nolan L, Harris JJ (2005). The in-vitro clinical failure of all-ceramic crowns and the connector area of fixed partial dentures: the influence of interfacial surface roughness. J Dent.

[14] Siegel SC, Patel T (2016). Comparison of cutting efficiency with different diamond burs and water flow rates in cutting lithium disilicate glass-ceramic. J Am Dent Assoc.

[15] Chang CW, Waddell JN, Lyons KM, Swain MV (2011). Cracking of porcelain surfaces arising from abrasive grinding with a dental air turbine. J Prosthodontics.

[16] Song XF, Ren HT, Yin L (2016). Machinability of lithium disilicate glass ceramic in vitro dental diamond bur adjusting process. J Mech Behav Biomed Mater.

[17] Nakamura Y, Hojo S, Sato H (2010). The effect of surface roughness on the Weibull distribution of porcelain strength. Dent Mater.

[18] Flury S, Peutzfeldt A, Lussi A (2012). Influence of surface roughness on mechanical properties of two computer-aided design/computer-aided manufacturing (CAD/CAM) ceramic materials. Operative Dent.

[19] Bagheri H, Hooshmand T, Aghajani F (2015). Effect of ceramic surface treatments after machine grinding on the biaxial flexural strength of different CAD/CAM dental ceramics. J Dent (Tehran).

[20] Benetti P, Della Bona A, Kelly JR (2010). Evaluation of thermal compatibility between core and veneer dental ceramics using shear bond strength test and contact angle measurement. Dent Mater.

[21] Phoenix RD, Shen C (1995). Characterization of treated porcelain surfaces via dynamic contact angle analysis. Int J Prosthodontics.

[22] Oh WS, Shen C, Alegre B, Anusavice KJ (2002). Wetting characteristic of ceramic to water and adhesive resin. J Prosthet Dent.

[23] Della Bona A, Shen C, Anusavice KJ (2004). Work of adhesion of resin on treated lithium disilicate-based ceramic. Dent Mater.

[24] Della Bona A (2005). Characterizing ceramics and the interfacial adhesion to resin: II- the relationship of surface treatment, bond strength, interfacial toughness and fractography. J Appl Oral Sci.

[25] Choi C, Driscoll CF, Romberg E (2010). Comparison of cutting efficiencies between electric and air-turbine dental handpieces. J Prosthet Dent.

[26] Lawson NC, Bansal R, Burgess JO (2016). Wear, strength, modulus and hardness of CAD/CAM restorative materials. Dent Mater.

[27] Wiegand A, Credé A, Tschammler C, Attin T, Tauböck TT (2017). Enamel wear by antagonistic restorative materials under erosive conditions. Clin Oral Investig.

[28] Rietveld HM (1969). A profile refinement method for nuclear and magnetic. J Appl Crystallogr.

[29] Coelho AA (2018). TOPAS and TOPAS-Academic: an optimization program integrating computer algebra and crystallographic objects written in C++. J. Appl. Cryst.

[30] Candido LM, Fais L, Ferreira EB, Antonio SG, Pinelli L (2017). Characterization of a diamond ground Y-TZP and reversion of the tetragonal to monoclinic transformation. Operative Dent.

[31] Aliaga R, Miotto LN, Candido LM, Fais L, Pinelli L (2020). Does diamond stone grinding change the surface characteristics and flexural strength of monolithic zirconia?. Operative Dent.

[32] Gorman CM, Horgan K, Dollard RP, Stanton KT (2014). Effects of repeated processing on the strength and microstructure of a heat-pressed dental ceramic. J Prosthet Dent.

[33] Tang X, Tang C, Su H, Luo H, Nakamura T, Yatani H (2014). The effects of repeated heat-pressing on the mechanical properties and microstructure of IPS e-max Press. J Mech Behav Biomed Mater.

[34] Al Mansour F, Karpukhina N, Grasso S, Wilson RM, Reece MJ, Cattell MJ (2015). The effect of spark plasma sintering on lithium disilicate glass-ceramics. Dent Mater.

[35] Ludovichetti FS, Trindade FZ, Werner A, Kleverlaan CJ, Fonseca RG (2018). Wear resistance and abrasiveness of CAD-CAM monolithic materials. J Prosthet Dent.

